# MHA-PINN: A Novel Physics-Informed Neural Network for Predicting Fiber Dyeability

**DOI:** 10.3390/s26072018

**Published:** 2026-03-24

**Authors:** Feier Zhou, Yuxiang Liu, Shuo Yang, Fan Guo, Xiaofeng Yuan, Ruimin Xie

**Affiliations:** 1 School of Information and Intelligent Science, Donghua University, Shanghai 201620, China; 230910209@mail.dhu.edu.cn (F.Z.); 2252310@mail.dhu.edu.cn (S.Y.); 2 College of Materials Science and Engineering, Donghua University, Shanghai 201620, China; 3 School of Automation, Nanjing Institute of Technology, Nanjing 211167, China; 4 School of Automation, Central South University, Changsha 410083, China

**Keywords:** physics-informed neural networks, variational autoencoder, domain knowledge, data augmentation, fiber dyeability

## Abstract

Fiber dyeability is a core indicator of textile quality and added value. Pre-experiment accurate prediction of fiber dyeability reduces the waste and inefficiency of trial-and-error methods. However, due to the limited data volume and complex mechanisms of fiber dyeability, there are no relevant studies to date. Thus, this paper proposes a novel prediction model integrating domain knowledge and process data called multi-head attention–physics-informed neural network (MHA-PINN). Within the MHA-PINN framework, limited experimental data is first augmented by using variational autoencoders, and subjected to ensemble feature selection on the augmented samples. Subsequently, a multi-head attention layer is introduced to capture the interdependencies among sample variables, thereby outputting a new feature matrix that represents the weighted fusion of these variables. Finally, a physics-informed neural network module embeds the dyeing kinetic equations into the loss function, guiding the model to converge towards accurate solutions for sample predictions. The effectiveness and superiority of the proposed MHA-PINN have been validated on a fiber dyeability experimental dataset.

## 1. Introduction

Fiber dyeability is a core indicator that directly determines the quality, added value, and market competitiveness of textile products. Accurate pre-experiment prediction of fiber dyeability can significantly reduce the waste of raw materials, energy, and time caused by the traditional trial-and-error method, which is of great significance for promoting the intelligent and green transformation of the textile industry. Among various fiber materials, Poly(propylene terephthalate) (PTT) stands out as a novel polyester polymer with outstanding comprehensive properties, such as good elasticity, chemical stability, and processability, and is widely used in bottles, clothing, packaging, sports and leisure goods, and household items [1,2,3,4].

Historically, research on polyester fibers has mainly focused on differential fiber preparation and functional modification, while studies on PTT and its derivative, cationically dyeable PTT (CDPTT), have primarily concentrated on their physical properties, e.g., thermodynamic and rheological behaviors, rather than dyeability prediction [5]. For example, Hsiao investigated the thermodynamic properties of PTT and CDPTT, confirming excellent interfacial adhesion between the polymers [6,7]. Yao et al. analyzed the rheological properties, thermal characteristics, crystallinity, toughness, and density of CDPTT [8]. In terms of dyeing process optimization, several studies have explored more ecological and economical routes by using harmless additives, such as environmentally friendly carriers, dispersants [9,10], and solvents [11] to achieve energy savings [12] and meet environmental protection requirements [13]. However, current research on CDPTT dyeability prediction remains scarce, and the industrial process for determining CDPTT dyeing performance is still complex and time-consuming. This not only increases labor and material costs but also fails to adapt to the demand for rapid process adjustment in intelligent production. More critically, CDPTT dyeing is a complex physicochemical process involving dye molecule penetration, diffusion, and adsorption, which is governed by multiple interacting parameters such as temperature, pH, dye concentration, and time. The limited availability of high-quality experimental data (due to high testing costs) and the nonlinearity of dyeing mechanisms further exacerbate the difficulty of establishing accurate prediction models. These challenges highlight the urgent need for a novel prediction method that can handle limited data and integrate complex physical mechanisms.

Traditionally, problems related to process prediction (including dyeability) have been modeled using ordinary differential equations (ODEs) or partial differential equations (PDEs), with analytical or approximate solutions obtained via numerical methods such as the finite difference method and finite element method. However, these methods often suffer from two drawbacks: first, they rely on strict simplifications of real-world mechanisms (e.g., ignoring the interaction between dye concentration and temperature), leading to deviations between theoretical and actual results; second, they are computationally expensive when dealing with high-dimensional or nonlinear systems. In recent years, physics-informed neural networks (PINNs) have emerged as a promising alternative, which is pioneered by Lagaris [14] and further developed by Raissi [15,16], Karniadakis [17] and others. PINNs embed physical laws (e.g., PDEs/ODEs) into the loss function, enabling them to learn from both data and domain knowledge. Notably, the fitting capability of PINNs does not directly depend on large-scale training datasets, their training process requires significantly less data than traditional deep learning models [18]. This ability makes them ideal for interdisciplinary scenarios with limited data, such as soft measurements and process prediction.

Existing applications of PINNs have demonstrated their effectiveness. For instance, Han’s team developed the model which is built on the methodology of a physics-informed neural network to predict the fatigue life of asphalt mixtures, supporting the design of durable road surfaces [19]. Prantikos applied the physics-informed neural network with the transfer learning method for transient prediction in nuclear reactors [20]. Furthermore, Hu et al. explored the use of PINNs in computational solid mechanics, analyzing their current capabilities and limitations [21]. Aji Nugroho et al. proposed that PINNs with temperature-corrected physical loss functions provide more accurate wellbore pressure predictions than conventional artificial neural network models [22]. Cong’s team incorporated residual compressive stresses into the physical model and integrated the physical model of crack growth rate with a neural network, significantly enhancing predictive accuracy compared to conventional physical models [23]. Song’s team proposed a mixed loss-guided modular regression framework, which incorporates physics-based modular decomposition and integrates a hybrid data–physics loss into the training, significantly enhancing predictive accuracy and computational efficiency compared to conventional regression methods [24]. Wang et al. further enhanced PINN performance in solving PDEs by introducing residual connections [25]. However, few studies have applied PINNs to fiber dyeability prediction, and existing PINN-based prediction models still have limitations in handling the issue of variable interdependency in CDPTT dyeing. The dyeing process is influenced by multiple correlated parameters (e.g., higher temperature accelerates both dye diffusion and hydrolysis), but traditional PINNs lack effective modules to capture such interdependencies, leading to suboptimal feature representation. Additionally, the problem of limited experimental data in CDPTT dyeing has not been addressed in existing PINN studies. The insufficient data may cause PINNs to converge to local optima, reducing prediction accuracy.

To address the aforementioned challenges which are limited data, complex variable interdependencies, and lack of physical mechanism integration in dyeability prediction, this paper proposes a novel multi-head attention–physics-informed neural network (MHA-PINN) that integrates domain knowledge (dyeing kinetic equations) and process data. The key design of the MHA-PINN framework is tailored to solve CDPTT dyeability prediction problems. Firstly, to alleviate the data scarcity issue, variational autoencoders (VAEs) are used to augment limited experimental data, and ensemble feature selection is performed on the augmented samples to eliminate redundant features (e.g., irrelevant auxiliary parameters) and improve model efficiency. Second, a multi-head attention layer is introduced to capture the nonlinear interdependencies among dyeing parameters (e.g., the coupling effect of temperature and time on dye uptake), outputting a weighted fused feature matrix that better represents the intrinsic relationship between input variables. Finally, a PINN module embeds dyeing kinetic equations (describing the diffusion and adsorption of dye molecules) into the loss function, guiding the model to converge toward physically consistent and accurate predictions.

This study is organized as follows. Section 2 presents the detailed architecture of MHA-PINN model. Section 3 presents the case study on the fiber dyeability, comparing the proposed MHA-PINN with other mainstream prediction models. Finally, the conclusions of the study are presented in Section 5, summarizing the key contributions, highlighting the significance of the proposed MHA-PINN model and outlining potential directions for future research.

## 2. Related Works

### 2.1. Instruments and Fiber Preparation

The raw material for preparing CDPTT was provided by Jiangsu Guowang High-Tech Fiber Co., Ltd. (Suzhou, China) in the form of chips, with an intrinsic viscosity [η] of 0.88 dL/g. Prior to melt spinning, these CDPTT chips were first subjected to vacuum drying at 110 °C for 24 h. Subsequently, rheological tests were performed using a Netzsch capillary rheometer of Malvern Instruments Ltd. (Malvern, UK) to verify that the chips met the technical requirements of the spinning process. After thorough drying and confirmation of rheological suitability, the CDPTT chips were fed into the hopper of the melt spinning equipment. Specifically, the melt spinning machine employed in this study was a POY-FDY (pre-oriented yarn and fully drawn yarn)-integrated melt spinning system, which comprised a single-screw extruder (Jiangsu Guowang High-Tech Fiber Co., Ltd., Suzhou, China [M12.1]) and an LHPQ 2-1000 drawing machine (Changzhou Lingxian Tetile Machinery Co., Ltd., Changzhou, China).

### 2.2. Data Preparation

**Figure 1:**
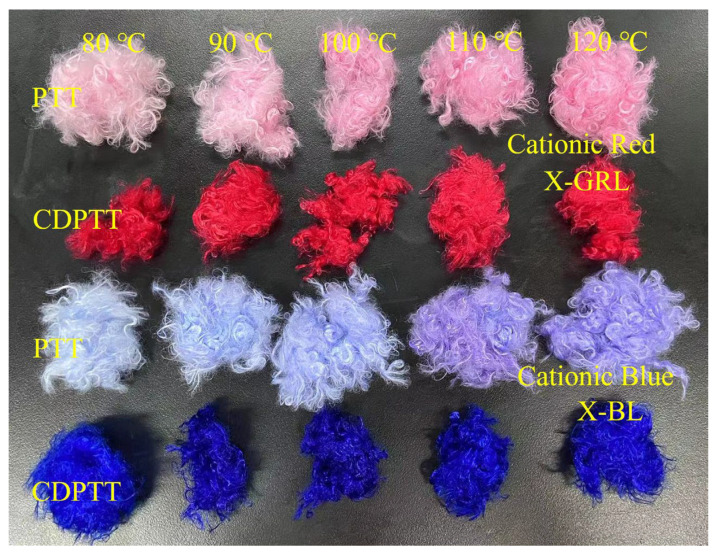
Physical images of CDPTT fibers and PTT fibers dyed under different dyeing condition [26].

To acquire sufficient and valid experimental data for model training and validation, forty sets of independent dyeing experiments were conducted, with five key process parameters varied to cover diverse operating conditions. Specifically, these experiments adopted a factorial design approach, wherein the five key process parameters, namely dyeing temperature, holding time, dye concentration, liquor ratio, and pH value, were adjusted within the ranges that satisfied constraints of spinnability. Based on practical dyeing feasibility and prior process experience, the parameter ranges were determined as follows: dyeing temperature must be controlled between 80 °C and 120 °C, holding time must be maintained between 20 min and 100 min, dye concentration should range from 1% to 5%, liquor ratio should adjusted between 1:20 and 1:40, and pH value must be regulated within 4 to 5. These ranges were confirmed to guarantee effective dyeing. The actual image of CDPTT samples after dyeing under representative conditions is shown in Figure 1.

Any deviation from the aforementioned parameter ranges was anticipated to result in suboptimal dyeing outcomes. After dyeing, the CDPTT specimens were rinsed to remove residual unabsorbed dye and then air-dried, thereby finalizing the entire dyeing process. Detailed parameters for all 40 experimental runs, including the five key variables and their specific values per trial, are listed in Table 1. In addition to the five key process parameters that need to be set, other auxiliary process parameters also required adjustment to ensure consistent experimental conditions. Since these parameters are not the focus of the next step of the study, they are not listed here individually.

Based on the aforementioned dyeing experiments, two key indicators of CDPTT dyeability, the dye uptake rate and K/S ratio, were measured for 40 samples. The dye uptake rate is calculated using the following Formula (1):

(1) $$E=\frac{A_{0}-A_{1}}{A_{0}}\times 100\%$$

where E represents the dye uptake rate (unit: %), $A_{0}$ denotes the absorbance of the original dye solution, and $A_{1}$ indicates the absorbance of the residual dye solution.

The K/S ratio is an important metric for characterizing the surface color depth of dyed fibers, defined based on the Kubelka–Munk theory (where K is the light scattering coefficient and S is the light absorption coefficient of the fiber). A higher K/S value directly corresponds to a deeper surface color of the CDPTT sample and a higher dye concentration adsorbed on the fiber surface.

To construct a high-precision dyeability prediction model, the original 40 sets of experimental data were insufficient. This is because deep learning models typically require a certain sample size to avoid overfitting and ensure generalization performance. Therefore, a well-established data augmentation method based on a variational autoencoder (VAE) model was employed to generate synthetic, physically consistent sample data. The 40 original experimental samples were randomly partitioned into training, validation and test sets in a 2:1:1 ratio. The variational autoencoder was trained exclusively on the 20 original training samples to generate 110 synthetic samples, all of which were incorporated into the training set. This yielded a final training set of 130 samples. After hyperparameter tuning on the validation set, the validation and test sets were combined into a single test set of 20 original samples for the final performance assessment.

### 2.3. PINN Deep Learning Method

Without loss of generality, generalized nonlinear ordinary differential equations can be expressed in the following form:

(2) $$\frac{du\left(t\right)}{dt}+N[u(t)]=0$$

where $u\left(t\right)$ denotes the Real-valued function, t  belonging to the intervals $\left[T_{0},T_{1}\right]$. N denotes a nonlinear differential operator that acts on u and can involve u and its derivatives up to order r.

Then using a neural network to approximate the solutions $u\left(t\right)$ as $\hat{u}\left(t;\theta \right)$, where θ denotes a set of network parameters. Substituting $\hat{u}\left(t;\theta \right)$ into the above equation, the partial differential equation residual is obtained as following

(3) $$f(t;\theta )≔\frac{d}{dt}\hat{u}\left(t;\theta \right)+N[\hat{u}\left(t;\theta \right)]$$

in automatic differentiation, computing the derivatives of $\hat{u}\left(t;\theta \right)$ to obtain f(t;θ). Then, optimizing network parameters f and training deep neural networks and residual networks gives

(4) $$Loss_{\theta }=Loss_{u}+Loss_{f}$$

and

$$Loss_{u}=\frac{1}{N_{IB}}\sum_{i=1}^{N_{IB}}{\left|\hat{u}\left(t_{IB}^{i};\theta \right)-u^{i}\right|}^{2},$$

(5) $$Loss_{f}=\frac{1}{N_{f}}\sum_{i=1}^{N_{f}}{\left|f\left(t_{f}^{i};\theta \right)\right|}^{2}$$

where ${\left\{t_{IB}^{i},u^{i}\right\}}_{i=1}^{N_{IB}}$ represents the initial value and boundary value training data points for sampling, while ${\left\{t_{f}^{i}\right\}}_{i=1}^{N_{f}}$ are the points sampled from the time domain for computing the residual f. $N_{IB}$ denotes the total number of boundary data points, while $N_{f}$ represents the number of residual points sampled within the computational domain.

## 3. Description of the Proposed Model

### 3.1. Data Preprocessing

To achieve high-precision and robust dyeability prediction, the original 40 sets of experimental data were deemed insufficient, as small datasets tend to cause deep learning models to overfit and compromise generalization performance. Therefore, the raw experimental data first underwent min–max normalization processing to eliminate the scale discrepancy among different process parameters, with the normalization formula defined as follows:

(6) $$x_{norm}=2\times \frac{x-x_{min}}{x_{max}-x_{min}}-1$$

where *x*_norm_ is the normalized value, x denotes the initial value of the variable, *x*_min_ and *x*_max_ represent the minimum and maximum values of all variables. After normalization, the newly normalized data were fed into the proposed model to execute the training procedure. Subsequently, the processed training data were then expanded to 130 samples by using a variational autoencoder (VAE), which maps the normalized samples and their corresponding conditional labels to the posterior distribution in the latent space.

**Figure 2:**
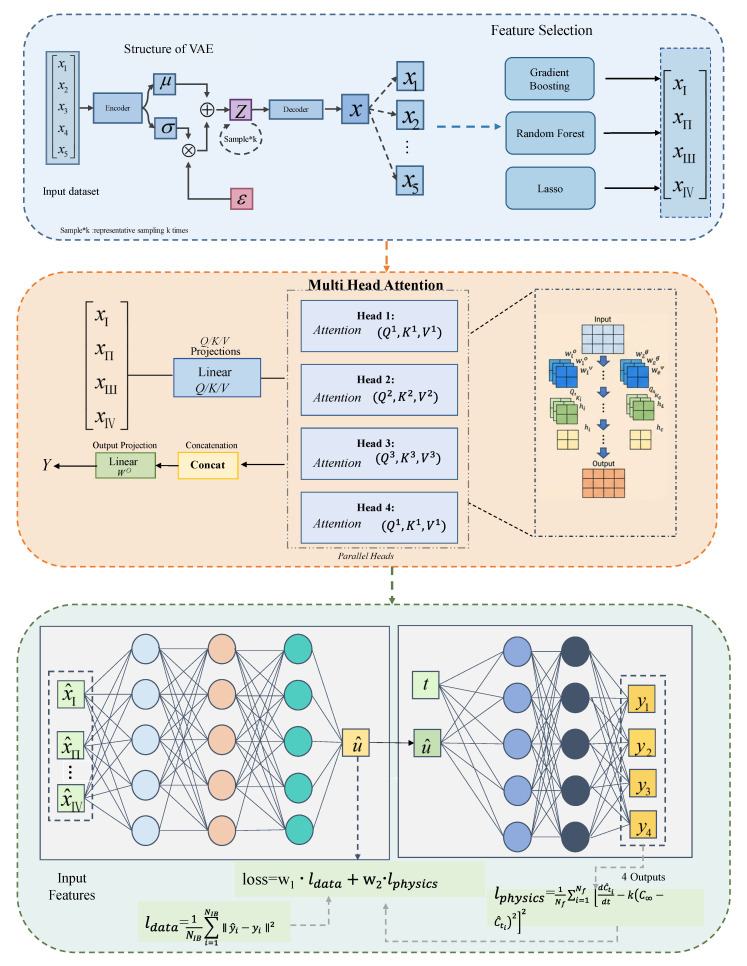
The structure of MHA-PINN.

To further optimize the model input and eliminate redundant features, an ensemble feature-selection model was employed to combine feature-selection methods with the principles of ensemble learning. Multiple feature-selection methods are integrated through specific rules. The ensemble feature-selection model adopted a heterogeneous ensemble architecture, where individual base learners were constructed using three distinct embedding-based feature-selection algorithms, specifically linear regression, random forest, and XGBoost. We set the number of trees in the random forest to 200 and the learning rate of XGBoost to 0.01. An ensemble rule is averaging, which takes the mean of the outputs from multiple feature-selection methods to mitigate the limitations of any single approach. The specific process of overall data processing and feature selection is shown in the green section of Figure 2.

**Figure 3:**
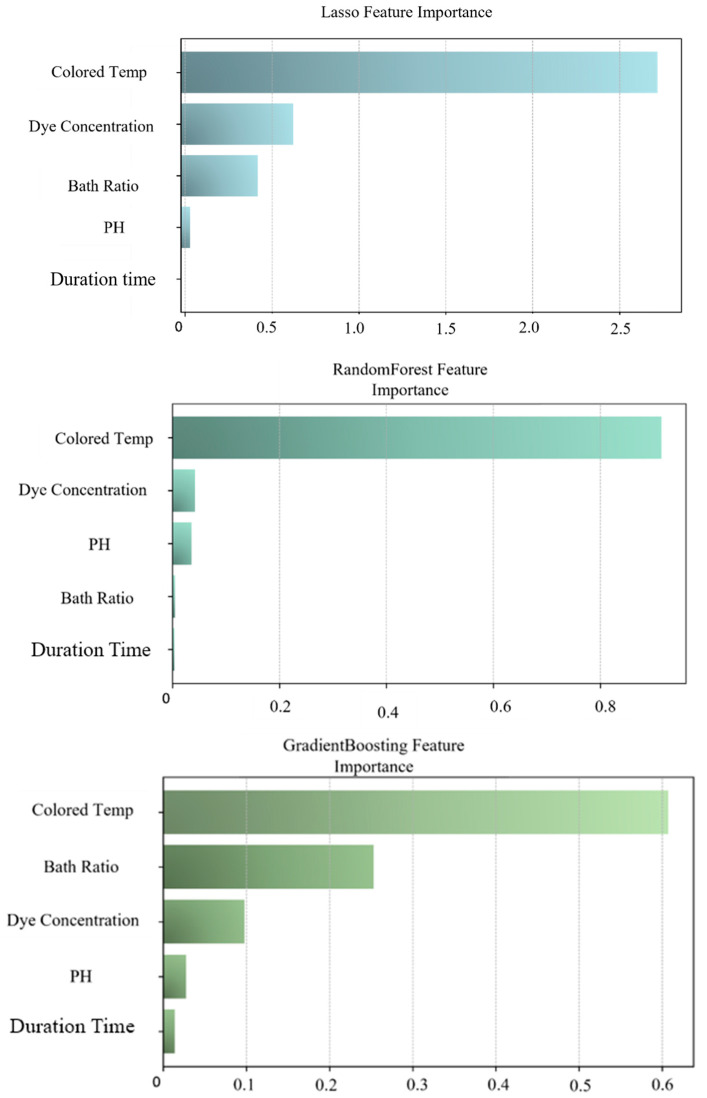
Schematic diagram of feature-selection results.

**Figure 4:**
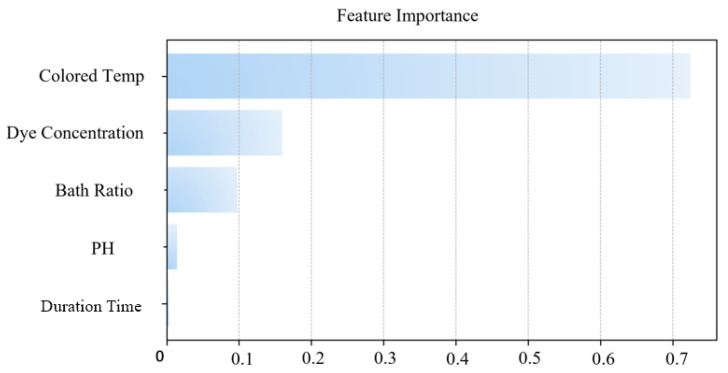
Schematic diagram of integrated feature-selection model results.

The three feature-selection methods were executed in parallel on the augmented dataset. The feature-importance scores from each method were normalized to a common scale, producing the results shown in Figure 3. The final integrated feature-importance scores were derived from the averaged values of the three normalized single-method scores, shown in Figure 4.

The importance scores of the final integrated feature-selection model are as follows.

The key insights from the integrated scores are summarized as follows: (1) the ‘time’ parameter received the lowest importance score and the ‘pH’ parameter received the second-lowest importance score, suggesting that the dye uptake process reaches an equilibrium state where prolonged time and pH value do not significantly enhance the outcome; (2) based on the integrated feature-importance ranking, three key parameters (dyeing temperature, dye concentration, and liquor ratio) were selected as the input variables for the subsequent dyeability prediction model. The following section details the physics-informed equation that still includes ‘time’ as an input variable, while the ‘pH’ parameter was excluded to reduce model complexity and computational cost.

### 3.2. Multi-Head Attention Mechanism

The attention mechanism originally emerged in the field of machine translation in machine learning. It aims to simulate the human ability to ‘focus’ when processing information, that is, to assign different weights to different positions of a given input sequence. Traditional single-head attention models achieve context modeling through a single Query–Key–Value operation; however, their expressive power is limited, making it difficult to capture multi-level interactions of information in different subspaces. Vaswani et al. [27] proposed MHA in the Transformer architecture, significantly improving the model’s representation and generalization capabilities. Given the representation X of an input sequence, X is first linearly mapped to obtain multiple Query, Key, and Value matrices, with the computation for the hth head shown in Equations (7)–(9):

(7) $$Q_{h}=XW^{Q_{h}}$$

(8) $$K_{h}=XW^{K_{h}}$$

(9) $$V_{h}=XW^{V_{h}}$$

where $W^{Q_{h}}$, $W^{K_{h}}$, and $W^{V_{h}}\in R^{d_{model}\times d_{k}}$ are the trainable parameters of the hth attention head and $d_{k}=d_{v}=d_{model}/H$, H is the number of attention heads. For each head, the scaled dot-product attention is calculated independently as shown in Equation (10):

(10) $$Head_{h}=Attention\left(Q_{h},K_{h},V_{h}\right)\;\;=softmax\left(\frac{Q_{h}K_{h}^{T}}{\sqrt{d_{k}}}\right)V_{h}$$

Then, after splicing the outputs of all heads, they are projected again to obtain Equation (11):

(11) $$MultiHead\left(X\right)=Concat\left(Head_{1},Head_{2},\ldots ,Head_{h}\right)W^{O}$$

$W^{O}\epsilon R^{d_{model}\times d_{model}}$ is the parameter matrix of the final linear transformation. We set $d_{model}$ to 16 and the number of attention heads h to 4. The core advantage of the multi-head mechanism is that each attention head in MHA can independently model different positional dependencies and features in a sequence, thereby improving the model’s generalization performance and stability in a time series. In this study, a multi-head attention module is introduced into the PINN model to enhance the model’s perception capabilities across different time scales and features.

### 3.3. PINN Architecture

The physics-informed neural network (PINN) incorporates domain-specific physical laws into neural network training by treating the residuals of governing physical equations as a part of the loss function. The core paradigm of PINNs lies in the joint optimization of two loss components: the data-driven prediction loss which quantifies discrepancies between model outputs and experimental observations and the physical constraint loss which penalizes deviations from known physical principles. This dual-objective optimization ensures that the network outputs not only fit the available experimental data but also adhere to fundamental physical laws, a key advantage for predictive tasks with limited data—such as CDPTT dyeability prediction in this study. As illustrated in the green part in Figure 2, the PINN architecture employed in this work consists of four core modules: input layer, hidden layer, output layer, and a physics-constrained loss function.

The input layer is used to input feature vectors extracted from four variables via a multi-head attention mechanism, where these variables are dyeing temperature, dye concentration, duration time value, and bath ratio. The first module is a fully connected feedforward neural network (FNN) comprising three hidden layers, with its architecture configured empirically through numerical simulations. This architecture choice is crucial for the model’s convergence and training efficiency. The model employs the rectified linear unit (ReLU) activation function, defined as

(12) $$f\left(x\right)=max\left(0,x\right)$$

Compared with traditional activation functions, ReLU offers two distinct advantages for PINN training. The first is that it maintains a constant derivative of one in the positive domain, which mitigates the gradient vanishing problem and ensures stable gradient propagation during backpropagation. The second is that its sparse activation mechanism (neurons with negative inputs are deactivated) reduces redundant computations, significantly improving the model’s training and inference efficiency.

To embed physical prior knowledge into the PINN framework, the second-order dyeing kinetic equation was incorporated into the loss function, ensuring model predictions comply with the intrinsic dynamics of the CDPTT dyeing process. Because the combination of CDPTT fibers and cationic dyes in this study is mainly driven by chemisorption, the pseudo-first-order kinetic equation cannot accurately describe the dyeing kinetics in this work. Therefore, we chose to introduce the second-order dyeing kinetic equation into the loss function as a physical prior, embedding it into the PINN framework to ensure that the model predictions align with the inherent kinetics of the CDPTT dyeing process. The second-order dyeing kinetic equation is expressed as

(13) $$\frac{dC_{t}}{dt}=k{\left(C_{\infty }-C_{t}\right)}^{2}$$

Under the assumption that the initial dye uptake is zero ($C_{0}=0$), the above formula can be integrated and transformed as follows:

(14) $$\frac{t}{C_{t}}=\frac{1}{C_{\infty }}t+\frac{1}{C_{\infty }^{2}\cdot k}$$

where *k* represents the second-order kinetic reaction rate constant, $C_{t}$ denotes the fiber staining rate at time *t*, and $C_{\infty }$ indicates the equilibrium staining rate. Regarding the parameter k and the parameter $C_{\infty }$, the equilibrium adsorption capacity $C_{\infty }$ and the reaction constant k can be calculated by fitting the slope and intercept of the curve according to the quasi-second-order kinetics. k is implemented as a trainable scalar parameter, with distinct values assigned to the cationic red and blue dyes to account for their different kinetic behaviors. Under the current experimental conditions, these rate constants are shared across all samples as global parameters, based on the assumption that the intrinsic kinetics remain consistent throughout the dataset. Experimental data show that under the dyeing condition of 100 °C, the adsorption process of both cationic dyes on CDPTT fibers follows a strong linear relationship, with $R^{2}$ values greater than 0.99. This result not only validates the applicability of the pseudo-second-order kinetic model to the system studied but also indicates that the pseudo-second-order kinetic equation is suitable for all dyeing conditions.

The overall loss function of the MHA-PINN model is a weighted sum of the data loss (*L_data_*) and the physical constraint loss (*L_physics_*), formulated as follows:

(15) $$\begin{array}{l} Loss=w_{1}\cdot l_{data}+w_{2}\cdot l_{physics}\\ =w_{1}\cdot \frac{1}{N_{IB}}\sum_{i=1}^{N_{IB}}∥{\hat{y}}_{i}-y_{i}{∥}^{2}+w_{2}\cdot \frac{1}{N_{f}}\sum_{i=1}^{N_{f}}{\left[\frac{d{\hat{C}}_{t_{i}}}{dt}-k{\left(C_{\infty }-{\hat{C}}_{t_{i}}\right)}^{2}\right]}^{2} \end{array}$$

where *w*_1_ and *w*_2_ are the weights for the two parts of the loss and, in this study, *w*_1_ is set to 1. As for $w_{2}$, we conducted a sensitivity analysis of the weight ranging from 10^−4^ to 1, accounting for changes in predicted RMSE values under different w_2_ scenarios. The results indicate that $w_{2}=0.001$ provides optimal balance between data fitting and physical constraint satisfaction.

The overall model structure is shown in Figure 2.

### 3.4. Evaluation of the Proposed Model

To comprehensively evaluate the prediction accuracy and generalization performance of the proposed MHA-PINN model, three widely used evaluation metrics were selected, respectively, the Root Mean Square Error (RMSE), Mean Square Error (MSE), and Coefficient of Determination ($R^{2}$). These metrics quantify different aspects of model performance, ensuring a holistic assessment. Their calculation formulas are defined as follows:

(16) $$RMSE=\sqrt{\frac{1}{N}\sum_{i=1}^{N}{\left(y_{i}-{\hat{y}}_{i}\right)}^{2}}$$

(17) $$R^{2}=1-\frac{\sum_{i=1}^{N}{\left(y_{i}-{\hat{y}}_{i}\right)}^{2}}{\sum_{i=1}^{N}{\left(y_{i}-\bar{y}\right)}^{2}}$$

(18) $$MSE=\frac{1}{N}\sum_{i=1}^{N}{\left(y_{i}-{\hat{y}}_{i}\right)}^{2}$$

where *N* denotes the sample size, ${\hat{y}}_{i}$ denotes the predicted values generated by the proposed PINN-based prediction model, $y_{i}$ denotes the target values, and y denotes the average of the target values. RMSE is a metric that measures the variation in predicted values around the target data. Higher accuracy is achieved when both RMSE and MSE values approach zero. The degree of fit between the predicted value and the target value is measured. When the value of $R^{2}$ approaches 1, higher accuracy can be achieved. To ensure equal contributions from each parameter in the proposed PINN-based prediction model, both the training and test sets should be normalized, while the output undergoes response normalization to obtain the final prediction results. This normalization is achieved through the normalization function.

## 4. Results and Discussions

### 4.1. Ablation Experiments

Ablation experiments were conducted to validate the effectiveness of each component within the MHA-PINN framework. Three primary approaches were employed in the experiments: the fully connected network (CN), the multi-head attention network (MHAN), and the physics-informed neural network (PINN). The CN is a baseline model, derived from MHA-PINN by removing both the multi-head attention mechanism and the physics-informed constraints. MHAN and PINN respectively represent models obtained from MHA-PINN by removing the physical information constraint layer and the multi-head attention layer. The hyperparameter determination method for all three models is analogous to that of MHA-PINN. Table 2 presents detailed results.

### 4.2. Comparative Experiments

To demonstrate the superiority of the proposed MHA-PINN in predicting CDPTT dyeability, we conducted a series of comparative experiments involving five models: the proposed MHA-PINN, which incorporates a multi-head attention mechanism and physical constraints from dyeing kinetic equations, and four benchmark models, including an artificial neural network (ANN), an extreme learning machine (ELM), mixed loss-guided modular regression (ML-MR), and symbolic regression (SR). The first comparative model is an ANN-based model. Compared to the proposed MHA-PINN model, the ANN lacks both the multi-head attention mechanism and physical constraints, while the ELM is a shallow model with no MHA, physical constraints, or complex feature engineering, the ML-MR model and the SR similarly omit the multi-head attention mechanism. All five models took the same four input variables, including dyeing temperature, dye concentration, duration time, and liquor ratio, and predicted four dyeability metrics, namely red up-staining rate, red K/S ratio, blue up-staining rate, and blue K/S ratio. The dataset first expands the 20 training samples to 130 samples using a variational autoencoder, and then the data is divided into a training set containing 130 samples and a test set with 20 samples to prevent data leakage.

Table 3 lists the RMSE, MSE, and $R^{2}$ values of the five models for predicting four dyeability metrics in both training and test sets. Equations (16)–(18) provide the definitions and interpretations of three evaluation metrics: RMSE, MSE, and $R^{2}$. Firstly, in the first row of each module, the predictive indicators for red over-staining rate are listed. For red dye uptake rate and red K/S ratio, the proposed MHA-PINN model achieved the smallest RMSE and MSE values, as well as the highest $R^{2}$ values, in both the training and test datasets. Further analysis reveals that MHA-PINN slightly outperforms the other four models. Since MHA-PINN’s $R^{2}$ approaches 1 while the ML-MR model deviates significantly from 1, MHA-PINN demonstrates marginal superiority over ANN and markedly outperforms ELM. For blue dye uptake rate and blue K/S ratio, MHA-PINN still achieves the highest $R^{2}\;$ values and the lowest RMSE and MSE values across both training and test datasets. Although the R^2^ value for blue dye uptake predicted by MHA-PINN was slightly lower than that achieved by SR, the proposed model consistently outperformed SR across the remaining three indices, yielding higher R^2^ values and superior scores on all other evaluation metrics. This result is mainly attributed to the MHA mechanism which captures nonlinear interdependencies between parameters and feature engineering from ensemble feature selection, as well as physical constraints that guide the model to avoid unphysical predictions.

**Figure 5:**
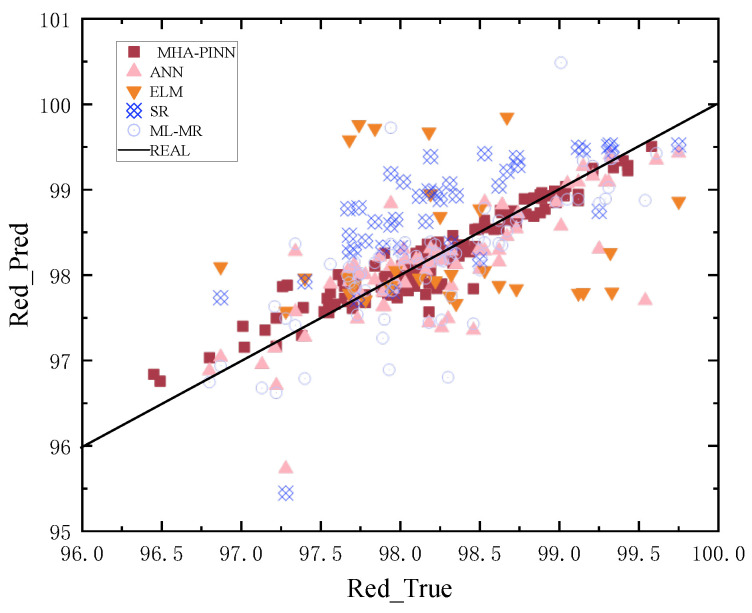
Predictions of five models for red dyeing rate.

**Figure 6:**
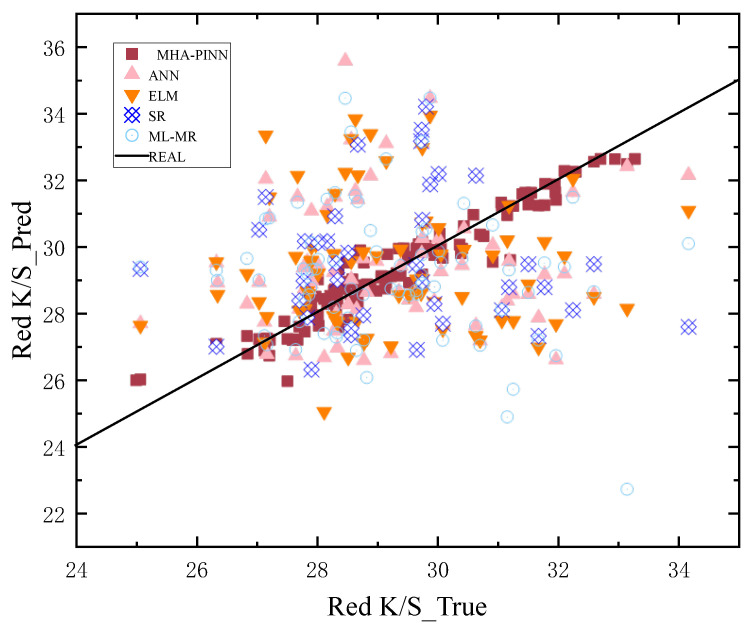
Predictions of five models for red K/S.

**Figure 7:**
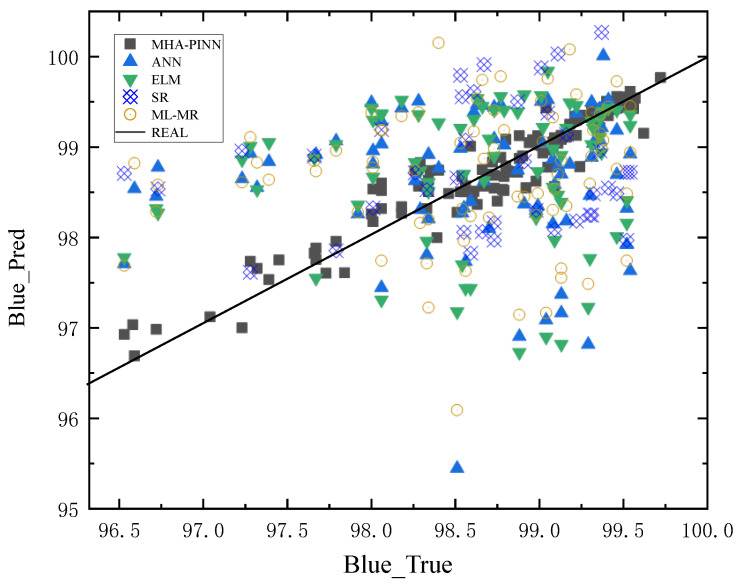
Predictions of five models for blue dyeing rate.

**Figure 8:**
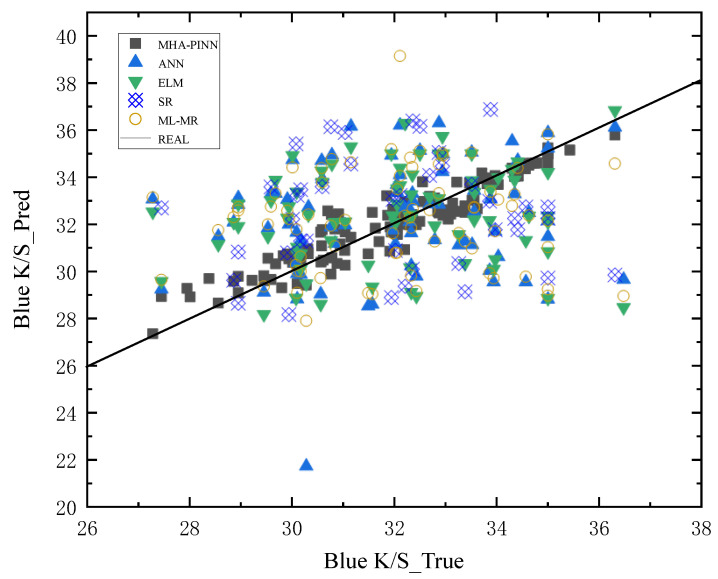
Predictions of five models for blue K/S.

To provide a more intuitive comparison, prediction scatter plots are shown in Figure 5, Figure 6, Figure 7 and Figure 8 below. In these plots, the x-axis represents the actual values of all samples, and the y-axis represents the predicted values for all samples. The black solid diagonal line denotes the ideal relationship between predicted and actual values. The closer the points are to the black solid line, the better the performance of the method. For the red dye uptake rate shown in Figure 5, it is evident that the square red dots representing MHA-PINN predictions are uniformly distributed on both sides of the standard line. In contrast, the predictions from the ELM (pink triangles), ANN (orange triangles), SR (blue squares), and ML-MR (light blue circles) models deviate substantially from the ideal line and exhibit non-uniform distributions, indicating their inferior predictive performance relative to MHA-PINN. Notably, ANN achieves relatively better predictive accuracy for red K/S compared to its performance on the other three indices, while SR demonstrates superior predictive capability for blue dye uptake rate relative to the other indices. Therefore, the scatter plots lead to an intuitive conclusion: the proposed MHA-PINN model exhibits superior predictive accuracy. Finally, given that these are multi-output models, their performance cannot be judged by a single prediction metric. Based on the combined evaluation of red and blue over-staining rates, along with the red K/S and blue K/S indices, the proposed MHA-PINN model is the optimal model for CDPTT dyeability prediction.

## 5. Conclusions

This study proposes an innovative deep learning model, MHA-PINN, which integrates the chemical properties of CDPTT dyeing processes with dyeing kinetic principles to predict CDPTT’s dyeing performance. Experimental results demonstrate that the combination of physical models and data-driven learning enables accurate prediction of CDPTT dyeing indices even with limited data under known operating conditions. To verify the effectiveness and superiority of the MHA-PINN model, comparative experiments were conducted with traditional models. In CDPTT dyeing performance prediction, the proposed MHA-PINN achieved an RMSE of 0.8411, significantly outperforming the basic artificial neural network whose RMSE is 1.2308, the mixed loss-guided modular regression whose RMSE is 0.9171, and the extreme learning machine ELM whose RMSE is 1.1959. However, this study also reveals a limitation of the current model. When dyeing conditions approach extreme states, e.g., temperature beyond 120 °C or pH outside 4–5, MHA-PINN prediction accuracy decreases and errors increase. Addressing this issue is crucial for improving the model’s prediction accuracy under all conditions, especially extreme ones. Future research should focus on refining the MHA-PINN architecture to enhance its predictive capability under extreme dyeing scenarios, thereby further expanding the model’s industrial applicability and reliability.

## Figures and Tables

**Table 1:** Forty sets of experimental data.

	Input Features					Labels			
Sample	Colored Temp (°C)	Duration (min)	Bath Ratio	Dye Concentration	pH	Red Up-Staining Rate (%)	Red K/S	Blue Up-Staining Rate (%)	Blue K/S
1	80	60	1:20	1%	5	96.45	28.31	96.53	27.45
2	85	60	1:20	1%	4	98.67	27.28	98.83	33.40
3	85	60	1:20	1%	4.5	98.7	28.71	99.07	33.62
4	85	60	1:20	1%	5	98.18	28.51	98.91	33.86
5	85	60	1:30	1%	5	97.26	28.11	98.34	31.49
6	85	60	1:40	1%	5	97.98	26.34	98.53	31.04
7	85	60	1:20	3%	5	99.25	33.95	99.39	35.74
8	85	60	1:20	5%	5	98.68	34.16	98.66	36.31
9	90	60	1:20	1%	5	98.12	30.38	98.16	33.38
10	95	60	1:20	1%	4	98.35	32.76	99.09	29.88
11	95	60	1:20	1%	4.5	97.17	32.26	98.79	30.7
12	95	60	1:20	1%	5	98.67	30.78	98.94	33.49
13	95	60	1:30	1%	5	97.9	30.63	98.54	30.08
14	95	60	1:40	1%	5	98.23	29.72	98.67	30.76
15	95	60	1:20	3%	5	99.29	33.85	99.43	34.27
16	95	60	1:20	5%	5	99.58	31.47	99.72	35.43
17	100	20	1:20	1%	5	98.14	28.69	99.58	31.14
18	100	40	1:20	1%	5	98.40	31.20	99.62	31.85
19	100	60	1:20	1%	5	98.45	32.57	99.68	34.18
20	100	80	1:20	1%	5	98.19	30.58	99.31	33.10
21	100	100	1:20	1%	5	97.89	28.60	99.09	31.32
22	100	60	1:30	1%	5	98.32	26.69	99.58	32.17
23	100	60	1:40	1%	5	98.19	24.99	99.24	27.95
24	100	60	1:20	3%	5	99.83	33.50	99.86	35.56
25	100	60	1:20	5%	5	99.75	33.36	99.84	35.30
26	105	60	1:20	1%	4	96.56	29.12	97.81	35.40
27	105	60	1:20	1%	4.5	97.78	29.04	97.44	36.30
28	105	60	1:20	1%	5	98.13	28.01	98.88	35.55
29	105	60	1:30	1%	5	97.72	27.50	97.54	30.61
30	105	60	1:40	1%	5	97.56	25.06	98.51	30.28
31	105	60	1:20	3%	5	98.69	30.70	99.04	36.48
32	105	60	1:20	5%	5	99.15	31.10	99.45	36.84
33	110	60	1:20	1%	5	92.03	28.64	94.31	25.85
34	115	60	1:20	1%	4	96.83	25.76	97.07	29.06
35	115	60	1:20	1%	4.5	97.78	26.83	97.32	29.53
36	115	60	1:20	1%	5	98.62	27.03	98.56	30.17
37	115	60	1:30	1%	5	98.06	26.32	97.79	28.95
38	115	60	1:40	1%	5	97.7	30.91	96.59	27.28
39	115	60	1:20	3%	5	99.54	30.21	99.49	33.99
40	115	60	1:20	5%	5	97.94	31.25	99.41	34.21

**Table 2:** Prediction results of CD-PTT for ablation experiments.

Models	Indexes	$R^{2}$	RMSE	MSE
CN	Red	0.740	0.336	0.113
	Red K/S	0.749	0.980	0.960
	Blue	0.430	0.537	0.289
	Blue K/S	0.769	1.035	1.071
MHAN	Red	0.663	0.396	0.157
	Red K/S	0.892	0.632	0.399
	Blue	0.546	0.515	0.265
	Blue K/S	0.788	0.952	0.907
PINN	Red	0.653	0.401	0.161
	Red K/S	0.890	0.632	0.399
	Blue	0.566	0.503	0.253
	Blue K/S	0.763	1.007	1.014
MHA-PINN	Red	0.801	0.386	0.149
	Red K/S	0.871	0.688	0.473
	Blue	0.813	0.439	0.193
	Blue K/S	0.834	0.841	0.707

Comparative results reveal that incorporating both the multi-head attention mechanism and the constraint layer led to varying degrees of improvement in the predicted $R^{2}$ values for all four outputs, alongside reductions in RMSE. These findings validate the effectiveness of both the multi-head attention layer and the physical information layer.

**Table 3:** Prediction results of CD-PTT for five methods.

Firstly	Indexes	RMSE	MSE	$R^{2}$
MHA-PINN	Red	0.3864	0.1493	0.8011
	Red K/S	0.6880	0.4730	0.8718
	Blue	0.4397	0.1934	0.8136
	Blue K/S	0.8411	0.7075	0.8343
ANN	Red	0.4289	0.1823	0.7231
	Red K/S	0.7482	0.5598	0.8427
	Blue	0.4817	0.2321	0.5939
	Blue K/S	1.2308	1.5148	0.6245
ELM	Red	0.4326	0.1870	0.5620
	Red K/S	1.8028	3.2501	0.0869
	Blue	0.5109	0.2610	0.5432
	Blue K/S	1.1959	1.4302	0.6455
ML-MR	Red	0.5213	0.2634	0.7225
	Red K/S	0.5019	0.2507	0.8127
	Blue	0.4931	0.2319	0.7894
	Blue K/S	0.9171	0.8453	0.7369
SR	Red	0.4506	0.2030	0.5187
	Red K/S	0.6273	0.3935	0.3185
	Blue	0.8032	0.6452	0.8217
	Blue K/S	1.1149	1.2430	0.7089

## Data Availability

All relevant data are contained within this paper.
